# The OMA orthology database in 2015: function predictions, better plant support, synteny view and other improvements

**DOI:** 10.1093/nar/gku1158

**Published:** 2014-11-15

**Authors:** Adrian M. Altenhoff, Nives Škunca, Natasha Glover, Clément-Marie Train, Anna Sueki, Ivana Piližota, Kevin Gori, Bartlomiej Tomiczek, Steven Müller, Henning Redestig, Gaston H. Gonnet, Christophe Dessimoz

**Affiliations:** 1 University College London, Gower Street, London WC1E 6BT, UK; 2 Swiss Institute of Bioinformatics, Universitätstr. 6, 8092 Zurich, Switzerland; 3 ETH Zurich, Computer Science, Universitätstr. 6, 8092 Zurich, Switzerland; 4 Institut National de la Recherche Agronomique (INRA) UMR1095, Genetics, Diversity and Ecophysiology of Cereals, 5 Chemin de Beaulieu, 63039 Clermont-Ferrand, France; 5 Bayer CropScience NV, Technologiepark 38, 9052 Gent, Belgium; 6 European Molecular Biology Laboratory, European Bioinformatics Institute, Wellcome Trust Genome Campus, Hinxton, Cambridge CB10 1SD, UK

## Abstract

The Orthologous Matrix (OMA) project is a method and associated database inferring evolutionary relationships amongst currently 1706 complete proteomes (i.e. the protein sequence associated for every protein-coding gene in all genomes). In this update article, we present six major new developments in OMA: (i) a new web interface; (ii) Gene Ontology function predictions as part of the OMA pipeline; (iii) better support for plant genomes and in particular homeologs in the wheat genome; (iv) a new synteny viewer providing the genomic context of orthologs; (v) statically computed hierarchical orthologous groups subsets downloadable in OrthoXML format; and (vi) possibility to export parts of the all-against-all computations and to combine them with custom data for ‘client-side’ orthology prediction. OMA can be accessed through the OMA Browser and various programmatic interfaces at http://omabrowser.org.

## INTRODUCTION

The flood of newly sequenced genomes presents a daunting interpretation challenge. Fortunately, the common origin of all living beings implies that many genes are conserved across species—in some cases despite billions of years of intervening evolution. Elucidating evolutionary relationships amongst genes and genomes is thus a key step in the analysis of new data. Sequences that have a common ancestry—homologs—are typically refined into orthologs, which are pairs of genes that started diverging via speciation, and paralogues, which are pairs of genes that started diverging via gene duplication (1,2). This distinction is useful in a broad range of contexts, including multigene phylogenetic inference, propagation of experimental knowledge from model organisms to non-model organisms and the study of gene evolution and adaptation (reviewed in 3,4). The need for orthology inference has led to the development of numerous methods (reviewed in 5) and databases, notably including EggNOG (6), Ensembl Compara (7), Inparanoid (8), MBGD (9), OrthoDB (10), OrthoMCL (11), Panther (12), PhylomeDB (13), Plaza (14) and OMA (15).

The OMA (Orthologous MAtrix) project is a method and database for the inference of orthologs amongst complete proteomes (i.e. the protein sequences associated for every protein-coding gene in all genomes). Initiated in 2004, OMA has undergone 17 major releases, steadily increasing the number of proteomes under consideration from 150 to 1706 across all domains of life. Besides its large scope, the distinctive features of OMA are the high specificity of the inferred orthologs (e.g. 16–19), feature-rich web interface, availability of data in a wide range of formats and interfaces and frequent update schedule of two releases per year.

In this update paper, after providing a brief review of the OMA pipeline, we present major new features recently added to OMA: a new web interface and reorganization, integrated gene ontology function prediction, better support of plant genomes, a synteny viewer depicting orthology relationships in their genomic context, statically computed hierarchical orthologous groups (HOGs) and the possibility to export genomes including all-against-all computations and to combine them with custom genome/transcriptome data.

## OVERVIEW OF THE OMA INFERENCE PIPELINE

OMA's inference algorithm consists of three main phases:

- First, to infer homologous sequences (sequences of common ancestry), we compute all-against-all Smith–Waterman alignments between every sequence and retain significant matches.
- Second, to infer orthologous pairs (the subset of homologs related by speciation events), mutually closest homologs are identified based on evolutionary distances, taking into account distance inference uncertainty and the possibility of hidden paralogy due to differential gene losses (20,21).
- Third, these orthologs are clustered in two different ways, which are useful for different purposes: (a) we identify cliques of orthologous pairs (OMA groups). Because all relations in one OMA group are orthologous, these are useful as marker genes for phylogenetic reconstruction and tend to be highly specific (18); (b) we identify HOGs, groups of genes defined for particular taxonomic ranges and identify all genes that have descended from a common ancestral gene in that taxonomic range (22).

OMA infers evolutionary relationships between genes from protein sequences, using one protein sequence per gene. If multiple splicing variants are possible, the best one in terms of matches with other genomes is selected, which is not necessarily the longest one (15).

## NEW WEB INTERFACE WITH BETTER ORGANIZATION

**Figure 1.**
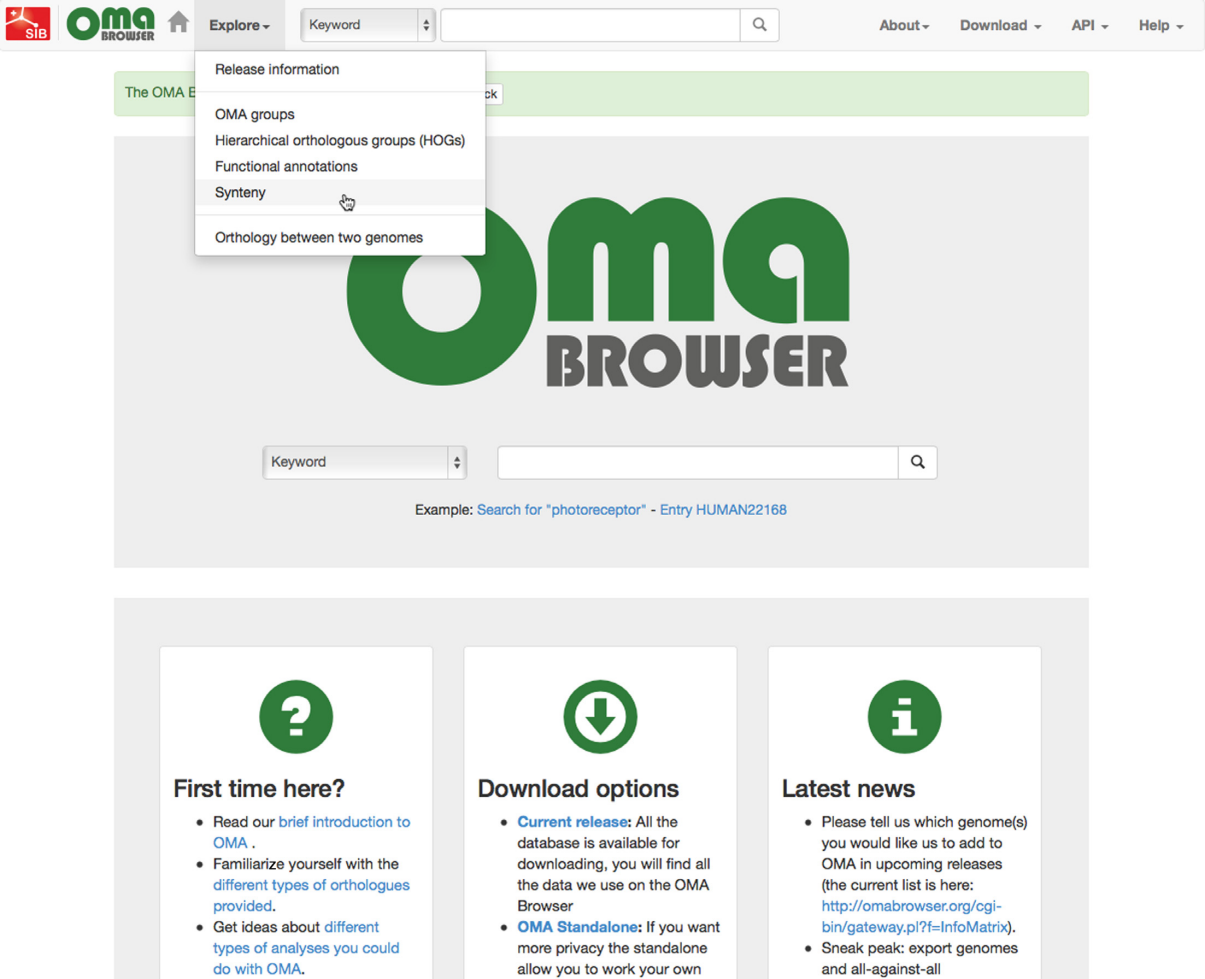
User-centric new design. The website has been redesigned with an emphasis on usability.

The OMA browser has been reorganised and redesigned to make it user-friendlier. The menu bar provides a consistent and persistent overview of all main functionalities. The documentation and help pages have been restructured and extended. The new ‘responsive’ layout takes advantage of large contemporary screens whilst also accommodating small screens such as smartphones and tablets. The landing page now provides pointers to introductory explanations for new users and recent announcements for returning users (Figure 1).

## GENE ONTOLOGY FUNCTION INFERENCE AS PART OF THE OMA PIPELINE

One key motivation for orthology inference is to computationally predict the roles that genes play in living organisms—e.g. Cellular Component, Molecular Function and Biological Process of the Gene Ontology (23). For many years, Gene Ontology (GO) annotations from the UniProt-GOA database (24) have been linked to all sequences in OMA. Additionally, we now provide inferred annotations based on orthology relationships: within the orthologous groups, we propagate GO annotations across different species.

**Figure 2.**
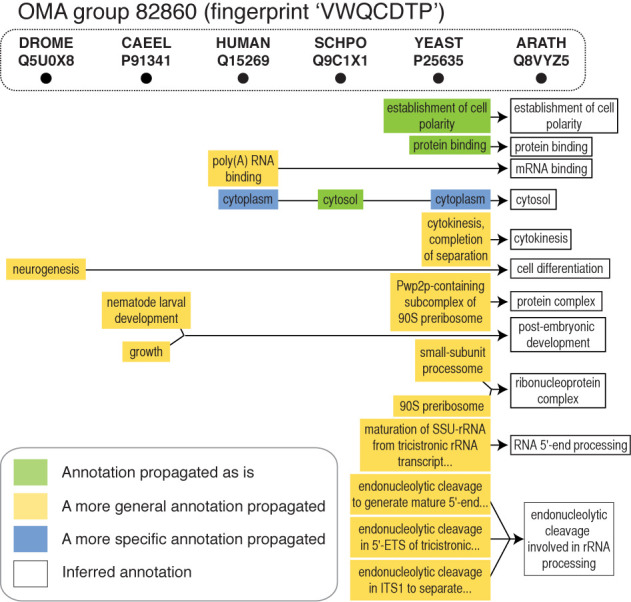
Gene Ontology propagation in the OMA pipeline. New Gene Ontology (GO) annotations for the sparsely annotated *Arabidopsis thaliana* protein Q8VYZ5 are inferred by propagating annotations from other members of the OMA group, taking into account implied parental terms and lineage-specific terms (see main text). For example, the inferred biological process Gene Ontology (GO) term ‘post-embryonic development’ is based on the more specific GO term ‘nematode larval development’; the latter is in itself inappropriate to assign to a protein in the plant clade. Proteins are labelled with their SwissProt/UniProt identifiers. The abbreviations ARATH, CAEEL, SCHIPO, DROME, HUMAN and YEAST refer to species *Arabidopsis thaliana, Caenorhabditis elegans, Schizosaccharomyces pombe, Drosophila melanogaster, Homo sapiens* and *Saccharomyces cerevisiae*, respectively.

To infer GO annotations, we start with curated annotations that are based on direct evidence from the literature: GO evidence codes EXP, IDA, IPI, IMP, IGI and IEP (http://geneontology.org/page/guide-go-evidence-codes). We then propagate them across OMA groups—sets of genes for which all members are inferred to be mutually orthologous—as these have been previously shown to be highly coherent in terms of functional annotations (25). Additionally, to avoid over-propagating clade-specific terms (e.g. ‘nematode larval development’ outside the nematodes), we require that propagated terms be used in at least one literature-based annotation in the clade in question. For example, the OMA group with fingerprint ‘VWQCDTP’ contains a *Caenorhabditis elegans* gene annotated with the GO term ‘nematode larval development’ (Figure 2); this term is not appropriate for genes outside of the Nematoda phylum. Therefore, when propagating this GO term to, for example, the poorly annotated *Arabidopsis thaliana* protein within the same OMA group, we only propagate those parent terms of ‘nematode larval development’ that are known to be associated with plant proteins; in this case, the most specific amongst those is ‘post-embryonic development’ (Figure 2). Indeed, the propagated annotation complements one of the known annotations for the *A. thaliana* protein, ‘embryo sac development’.

**Figure 3.**
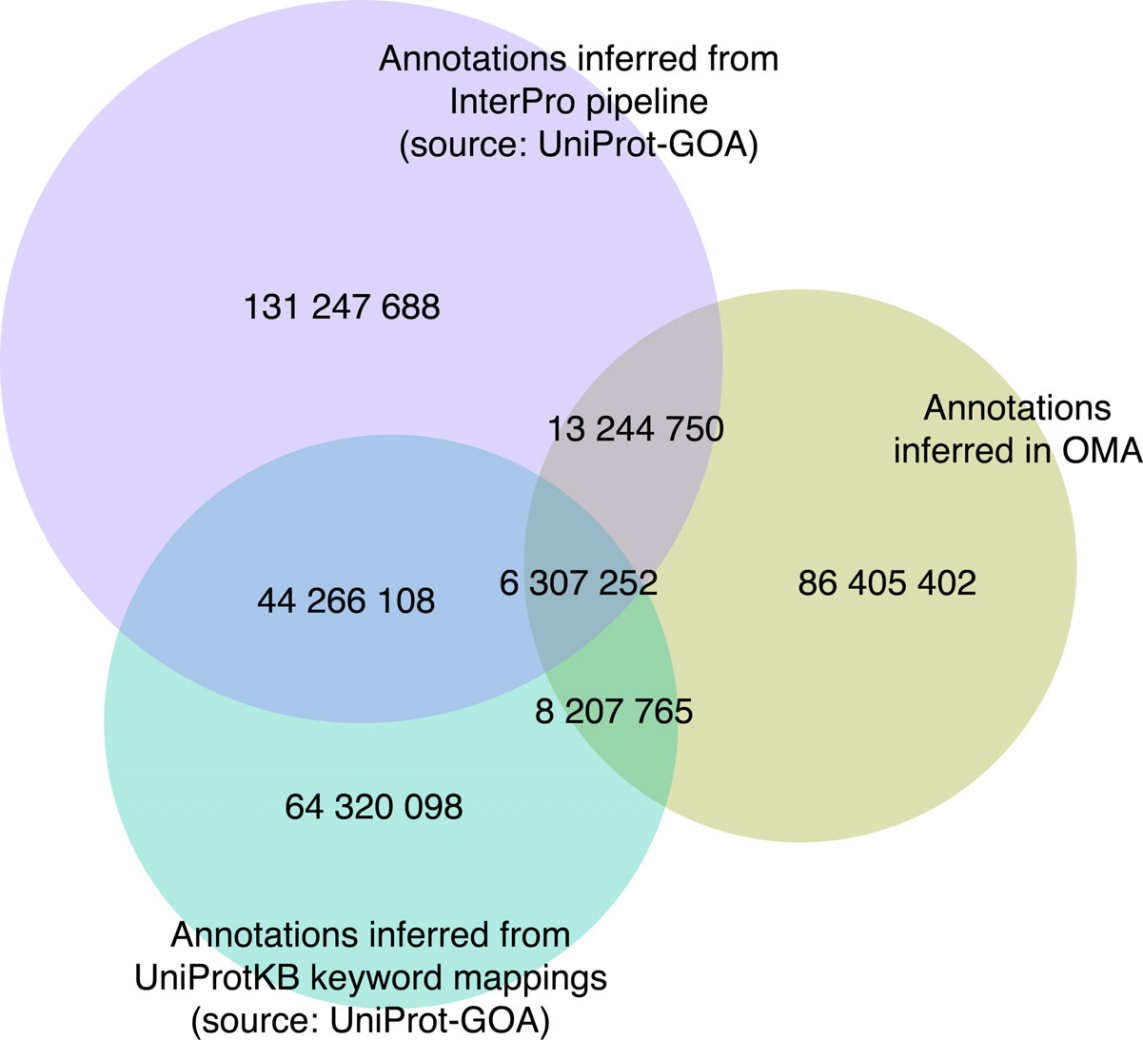
Numbers of electronic Gene Ontology annotations in the OMA database. Three major sources of electronic annotations are shown: annotations through the association of InterPro records with GO terms, annotations based on UniProtKB keyword mappings and annotations inferred in the OMA pipeline. The intersections show the numbers of annotations in common amongst the resources.

Overall, the OMA database now provides 442 376 477 function annotations for 7 947 728 proteins (Figure 3). Amongst the available annotations, most are computationally inferred; our own predictions constitute about 20% of the available annotations.

Function annotations based on OMA orthologs are particularly valuable for proteins for which other computational annotation methods provide no annotations and the available annotations assigned by curators are relatively general and/or sparse. In the most recent OMA release, we provide annotations for 423 983 proteins for which there are no other electronic annotations. For example, at the time of writing the *A. thaliana* protein with UniProt identifier Q8VYZ5 had no electronically inferred GO annotations (evidence code IEA); it had five annotations based on evidence codes ISS or RCA, which are not used in our propagation pipeline; and the annotations from literature-based evidence were ‘nucleolus’ (IDA), ‘rRNA processing’ (IMP) and ‘embryo sac development’ (IMP). Using our OMA annotation pipeline, we assigned new annotations that complement these: for example, we inferred GO terms ‘RNA 5’-end processing’ and ‘endonucleolytic cleavage involved in rRNA processing’ that complement the known experimental annotation ‘rRNA processing’; we inferred the GO term ‘post-embryonic development’ that complements the known experimental annotation ‘embryo sac development’ (Figure 3).

## BETTER SUPPORT FOR PLANT GENOMES, INCLUDING HOMEOLOGY IN WHEAT

One research area where comparative genomics can make an important difference is modern crop science. Indeed, plant genomes tend to have highly redundant genomes as a result of their complex history of duplication and hybridisation events. With almost all genes being available in several copies on multiple sub-genomes, the use of comparative genomics is essential in order to map knowledge across different species. Several specialised plant resources already exist—such as Ensembl Plants (26), Gramene (27), Greenphyl (28) and Plaza (29)—but there is value in providing plant support in resources inferring orthology across all domains of life. Also, plant-based analyses can benefit from the other distinctive features of OMA, such as its highly specific predictions and ability to infer HOGs. We have improved plant genome support in OMA by adding and updating more plant genomes and by inferring and annotating homeology—genes related through polyploidization—in the wheat genome.

The number of plant species in the OMA database has increased from 8 to 28 plants in recent years. In the latest release, we have added *Selaginella moellendorffii* (a lycophyte) as the deepest branching vascular plant and *Physcomitrella patens* (a bryophyte) as a representative of the non-vascular plants, thus widening the taxon set to cover ∼450 million years of plant evolution (30). We have also added the important model grass species *Brachypodium distachyon* and *Aegilops tauschii*. Additionally, we have added a variety of crop species of practical and economic importance, which are especially useful to plant geneticists and breeders. These species include: banana (*Musa acuminata subsp. malaccensis*), potato (*Solanum tuberosum*), several rice species (*Oryza brachyantha*, *Oryza glaberrima*, *Oryza sativa subsp. indica*), foxtail millet (*Setaria italica*) and bread wheat (*Triticum aestivum*).

In particular, bread wheat is the staple food source for 30% of the human population, making it one of the world's most important cereal crops. However, its very large (17 Gb), highly repetitive, hexaploid (2n = 6x = 42) genome, has made studying its organization and evolution notoriously challenging due to the lack of a high-quality reference sequence. Wheat is a recent allopolyploid resulting from two recent (<0.8 MYA ago) hybridization events between three diploid progenitors, of which the most distant pair diverged an estimated 6.5 MYA ago (31). Following that hybridization event, there has seemingly been little or no recombination across the chromosomes derived from the three progenitor genomes (32). It is therefore helpful to think of these three sets of chromosomes as ‘subgenomes’. This gives rise to the notion of homeologous (also spelled ‘homoeologous’) chromosomes—closely related pairs of chromosomes between two subgenomes. These homeologous chromosomes have maintained a high degree of conservation amongst them, with highly similar genes located on the same chromosomal group (1 to 7) of each subgenome. However, because there have been extensive gene duplications, losses and rearrangements in the *Triticeae* lineage (32–35), the relationship across homeologs is not necessarily 1:1:1.

In OMA, we define homeologous genes as pairs of homologous genes that have started diverging through speciation between the progenitor genomes and then merged back into the same genome by hybridization. Thus, homeologs can be thought of as ‘orthologs between subgenomes’. This suggests a simple way of adapting the OMA pipeline to infer homeologs: we first partitioned the predicted wheat proteins into the three subgenomes based on the annotation of the IWGSC (32), then inferred ‘orthologs’ between these subgenomes using our standard pipeline. Although conceptually straightforward, this procedure is complicated by the fragmentary nature of the current wheat survey genome, consisting of many contigs and resulting in numerous genes which are split, misannotated, or simply missing.

Dubious homeolog inferences are discarded in two steps. The first filter, part of the standard OMA algorithm, identifies instances of differential gene losses through witnesses of non-orthology in a third genome (21). This filter discarded 4166 pairs. The second filter, developed specifically for homeology detection, considers the distribution of the evolutionary distances and removes outliers (defined as gene pairs with a distance higher than 2.5 standard deviations above the mean distance) from the set of reported homeologs. This discarded an additional 2212 pairs.

**Figure 4.**
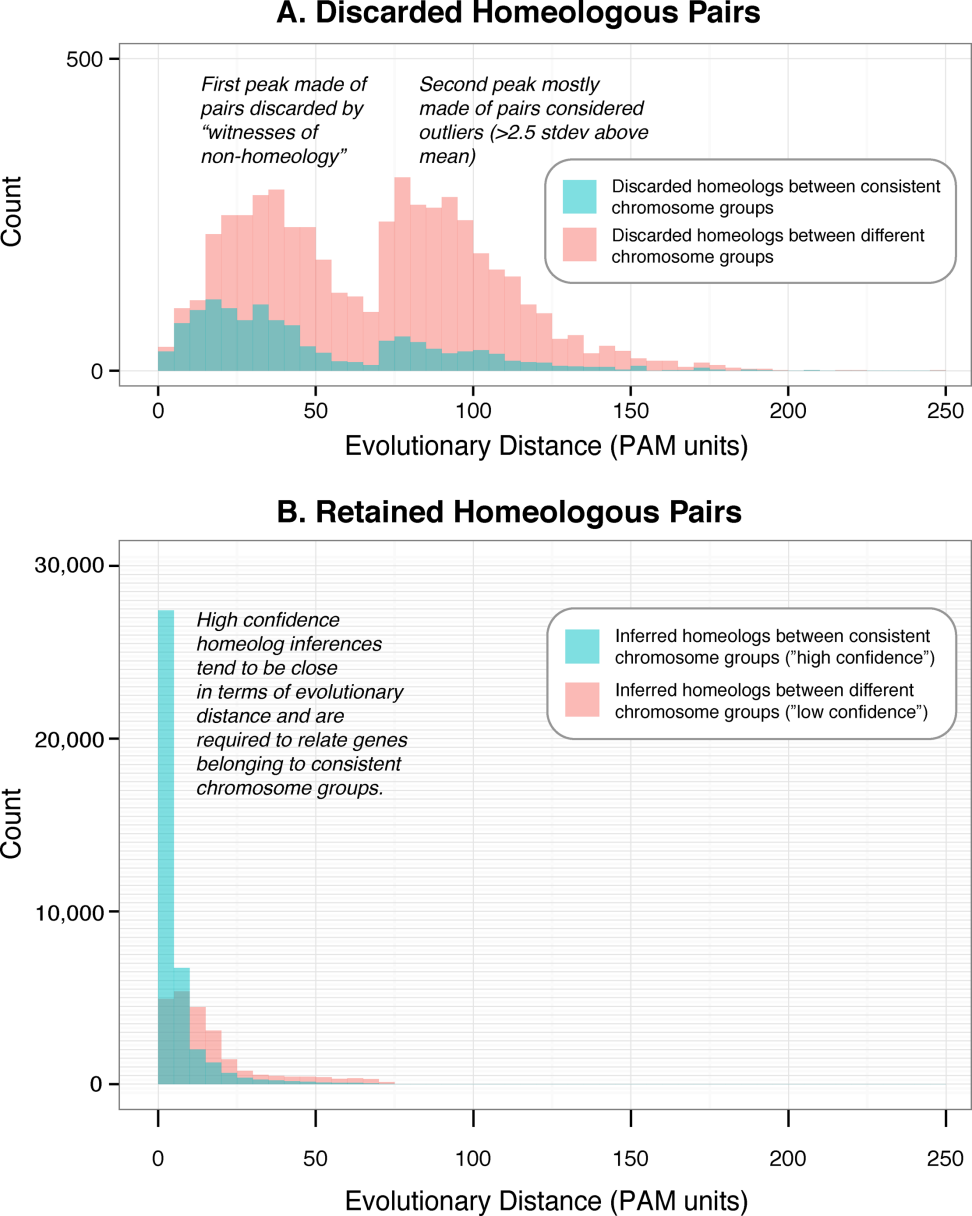
Distribution of evolutionary distances for homeologous pairs that were (**A**) discarded via witness of non-homeology or because they were outliers, or (**B**) retained as inferred homeologs. In both plots, the blue colour represents pairs where both homeologs are located on the same chromosome group and the red colour indicates pairs where homeologs are located on different chromosome groups. The y-axes are drawn at different scales but the grid is consistent across the two plots.

Two indicators suggest that the bulk of these discarded pairs are indeed unlikely to be homeologous. First, assuming that the majority of genes have remained in their ancestral position in the *Triticeae* lineage, most homeologous relationships should be between genes on corresponding chromosome groups. Yet only 14.7% of all the pairs discarded by witnesses of non-orthology and 34.7% of outliers are inferred to be between the same chromosome group (compared to 14.5% for random pairs). Second, because the three progenitor genomes diverged relatively recently (∼6.5 MYA), most homeologs can be expected to be highly similar. Yet the evolutionary distance between discarded homeologous pairs is on average much higher than for the retained pairs, even if we only consider pairs filtered in the first step (Figure 4A).

We applied the same indicators to the 62 910 retained homeolog inferences. The proportion of retained homeologs involving pairs of genes on corresponding chromosome groups was considerably higher (62.8% versus 14.7–34.7% for discarded pairs). Furthermore, as expected, the distribution of evolutionary distance between predicted homeologs was skewed towards low distances, with a mean of 12.6 PAM (0.126 substitutions per site) and a standard deviation of 20.6 PAM (Figure 4B). As an additional assessment, we selected a random subset of 20 homeologous gene pairs and performed a manual validation taking into account sequence quality, gene annotation, shared chromosome group, percentage identity and evolutionary distance between pairs. Fifty-five percent of the predictions could be confirmed, with the rest being either inconclusive or likely mistakes due to misannotations (transposons, chloroplast genes), missing true homeologous counterparts, etc. (Supplementary Table S1). Given that the process of flow sorting of the wheat chromosomes and arms resulted in on average 10% contamination with other chromosomes (32), a small proportion of bona fide homeolog pairs can be expected to be erroneously annotated as belonging to different chromosome group.

In the OMA browser, retained homeolog inferences are labelled as ‘high confidence’ if they involve genes belonging to consistent chromosome groups, and ‘low confidence’ if they do not. In the latest release, this resulted in 39 442 pairs (63.2%) of high-confidence homeology predictions and 23 468 (36.8%) low-confidence ones. The average percent identity for the 12 high confidence pairs is 95.4% compared to 90.5% for low confidence pairs. We chose not to be too stringent in the cut-off for evolutionary distance and/or percent identity because although most homeolog pairs have a high degree of conservation, this might not necessarily be true for certain genes that evolve quickly such as disease resistance genes (36), transcription factors (37) or pentatricopeptide repeat proteins (38).

## NEW SYNTENY VIEWER PROVIDING THE GENOMIC CONTEXT OF ORTHOLOGS

In the absence of genome rearrangement, orthology relationships can be expected to be consistent across neighbouring genes—a concept commonly referred to as ‘shared synteny’. Patterns of syntenic conservation or divergence can shed light on the evolutionary history of genomic loci of interest; they can also reveal sequencing artefacts, misannotations or orthology inference errors. Synteny visualization tools have been successfully developed in several comparative genomics databases such as Yeast Gene Order Browser (39), Genomicus (40) or GnpIS (41). The OMA Browser now features a synteny viewer as well.

The OMA synteny viewer uses a typical layout: genes are represented by boxes, with neighbouring genes displayed in adjacent columns and orthologous regions displayed in different rows. The reference syntenic block, centred on a query gene, is displayed in the first row. The other rows are centred on genes that are orthologous to the query gene, ordered by increasing taxonomic distance to the query gene species. Orthology relationships to each gene contained in the reference syntenic block are coded using different colours. To convey many-to-one and many-to-many relationships, we use stripes of the relevant colours. To aid clarity, hovering over a gene highlights all orthologs of the same colour including those with stripes. The data can be conveniently explored by clicking on any gene, which recentres the display on that gene as a new query.

**Figure 5.**
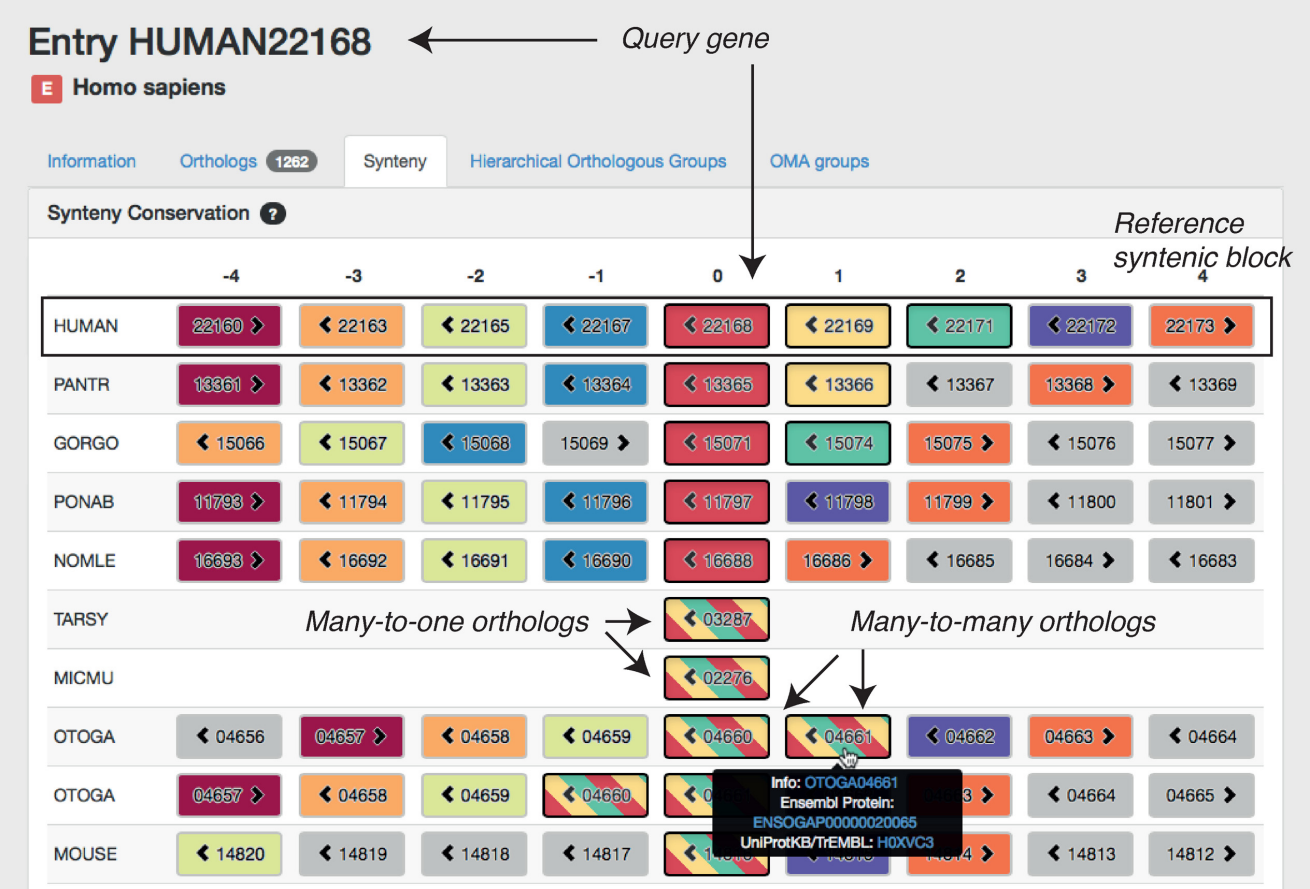
Screenshot of the new OMA synteny viewer with the *ADH1A* gene in human (Gene ID 22168) as query. Each gene is illustrated as a box containing a numerical OMA Gene ID and an arrow to indicate the gene's orientation. The colour of genes outside the query species indicates orthologous relationship with human genes, with bands of colour capturing many-to-one and many-to-many relationships. Genes that are non-orthologous to all nine human genes contained in this window are displayed in grey. The fragmented assemblies of tarsier (TARSY) and mouse lemur (MICMU) contain no genes next to 03287 and 02276, respectively.

To illustrate the usefulness of the new synteny viewer, consider the arrangement of alcohol dehydrogenase (ADH) genes around human *ADH1A* (Figure 5). The human ADH gene cluster ADH7 (class IV)-ADH1C (class I)-ADH1B (class I)-ADH1A (class I)-ADH6 (class V)-ADH4 (class II)-ADH5 (class III) is displayed in the first row. Because the cluster sits on the complementary strand, it appears in reverse order—starting in column 3 (Gene ID 22172) and ending in column −3 (22163). The synteny viewer suggests that the neighbourhood of orthologous genes is well conserved amongst simians, but the conservation diminishes as we move to more distant lineages. Genes with stripes are in one-to-many or many-to-many orthologous relationships with human ADH1A (22168), human ADH1B (22169) and human ADH1C (22171). In particular, the presence of two orthologs in the bushbaby (OTOGA) suggests a separate duplication within the lemur lineage, yielding many-to-many orthology. These observations are all consistent with detailed analyses in the literature (42). Although positioned within well-conserved syntenic regions, genes 13367 in the chimp (PANTR) and 15069 in the gorilla (GORGO) have no human orthologous counterpart in this region. On account of their very short lengths—13 AA and 14 AA, respectively—they are likely to be fragments. Furthermore, the absence of flanking genes in the tarsier (TARSY) and mouse lemur (MICMU) is due to the low quality of the genome assembly in these regions.

## BETTER SUPPORT FOR HOGs

As discussed above in the overview of the OMA pipeline, HOGs are a key output of the OMA algorithm; they group all the sequences that have descended from a single common ancestral gene within clades of interest. This provides an intuitive framework to generalise the concept of orthology to more than two species. For instance, if we consider the human ADH1A gene discussed in the previous section, it belongs to an HOG containing ADH1B and ADH1C as well, whilst at the more specific level of simians, the three genes belong to three distinct HOGs. This difference in resolution makes intuitive sense because as we consider a broader or narrower range of species, the shared attributes amongst them can be expected to be coarser or finer.

OMA HOGs are inferred from orthologous pairs using a fast and effective algorithm described previously (22). However, until recently, the OMA Browser had been dynamically inferring these HOGs on user demand. Large families could take a few minutes to process. Furthermore, because of the non-deterministic nature of the inference algorithm, there could be small inconsistencies for requests at different taxonomic levels (e.g. one sequence included in an HOG defined at the level of vertebrates but not included at the level of all bilateria). Starting with the latest release, HOGs are precomputed thereby providing rapid user access and consistent inferences. HOGs can now be downloaded in OrthoXML format (43) for further analyses.

One potential use of the HOGs data is to map gene losses, duplications and gains onto species trees. Indeed, since HOGs are defined in terms of ancestral genomes at all internal nodes in the species tree, keeping track of the number of HOGs and their content whilst traversing the tree can yield these quantities. Contrary to approaches solely based on gene counts in extant genomes (e.g. 44), HOGs take into account relationships between the actual sequences and thus can be expected to yield more precise estimates. Furthermore, this approach allows the user to identify the specific genes that underwent duplication or losses on particular branches of the phylogeny.

**Figure 6.**
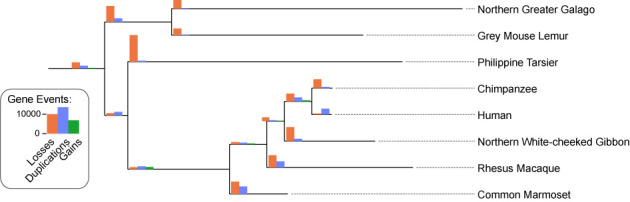
Gene losses, duplications and gains from hierarchical orthologous groups. Gene duplications, losses and gains on the primate lineage inferred from OMA hierarchical orthologous groups.

To illustrate this application, we provide an estimate of gains and losses in the primate tree obtained by parsing OMA HOGs (Figure 6). Large numbers of losses on terminal branches can be indicative of fragmentary genomes (45), such as the tarsier with its low 1.82x coverage. Even so, previous studies have reported elevated duplication and loss rates in the primate lineage (46).

**Figure 7.**
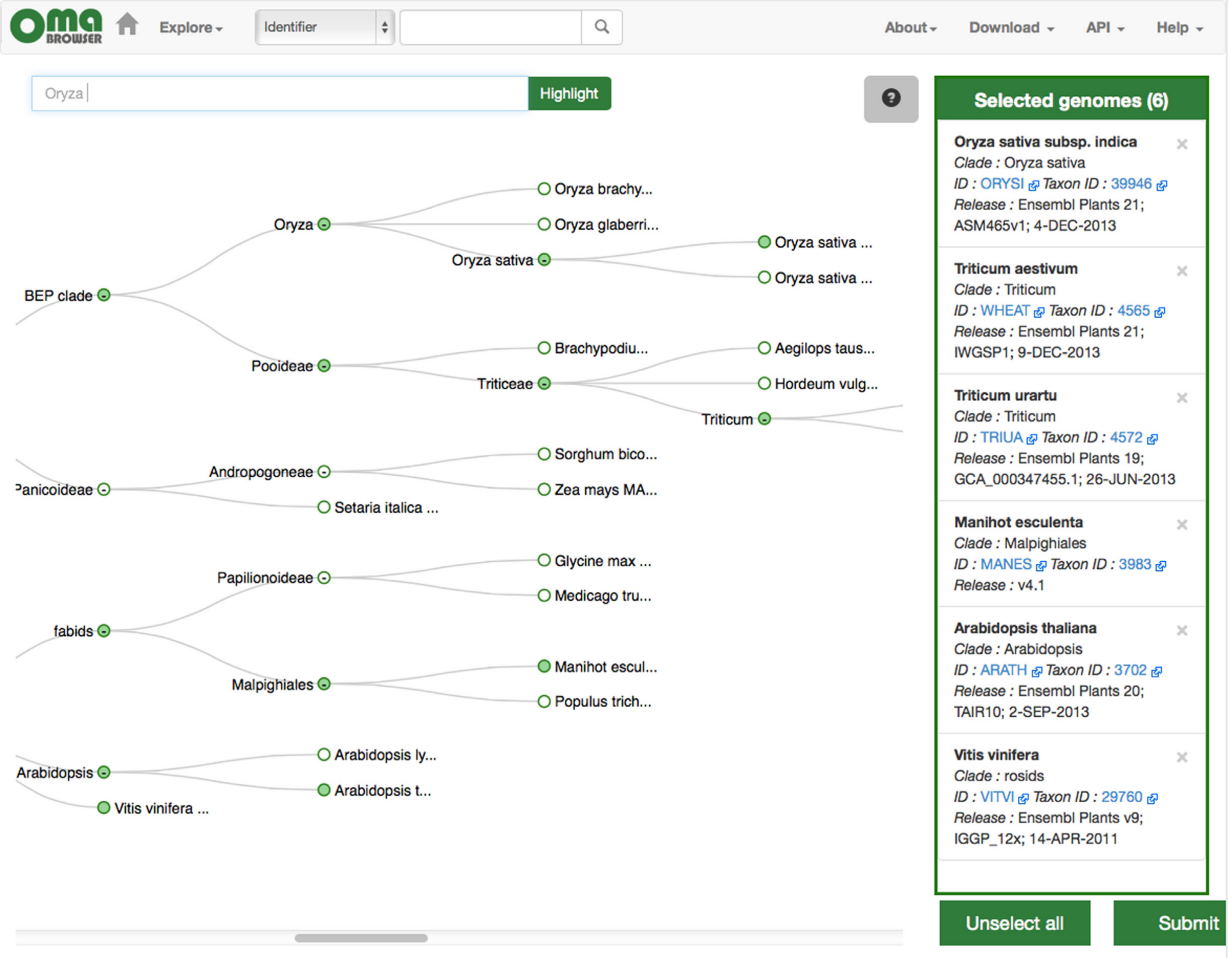
Selection tool for pre-computed genome export. This new function enables users to export genomes of interest and their associated all-against-all comparisons for analysis in the OMA standalone software.

## EXPORT OF PROTEIN SETS AND THEIR ASSOCIATED ALL-AGAINST-ALL COMPUTATIONS

As genome and transcriptome sequencing are becoming affordable and ubiquitous, there is an increasing need for orthology prediction on custom data. As a solution to this, we have developed OMA standalone, a downloadable open source implementation of the OMA pipeline for Linux and Mac (the details of the software are the focus of a forthcoming publication). To enable users to efficiently combine custom and public genomes, we have added the possibility of exporting OMA genomes, including all-against-all computations amongst them, as input files for OMA standalone. The function is accessible via the ‘Download’ menu in the navigation bar of the new OMA Browser interface. Users can select up to 50 genomes for export (Figure 7), which together with OMA standalone are packaged for download as a single compressed *tar* file.

## OUTLOOK

For just over a decade, the OMA database has provided orthology inference amongst complete genomes. It has remained true to its mission of providing reliable, high-quality orthology inferences across a broad taxonomic range. With 17 major releases, each including ∼100 additional and updated genomes, the project has been maintained with sustained endurance. At the same time it has also gained numerous functionalities, of which the most recent are highlighted in this update.

So what awaits OMA in the coming decade? One major challenge facing many phylogenomic resources is to keep abreast of the rapid increase in sequencing data (4). In OMA, the all-against-all protein comparison phase—the most time-consuming phase with >7 million CPU hours logged to date—grows quadratically with the number of sequences under consideration. But computational bottlenecks are nothing new in OMA; they have been a *leitmotif* all along and our experience has been that they can generally be overcome through software optimization (e.g. 47) or new heuristics (e.g. 48). We also see potential in sharing computations across different resources and have initiated a joint effort with OrthoDB (10) in that direction.

Another challenge lies with fragmentary, poorly annotated genomes and their potentially disruptive effect on orthology inference and interpretation. Yet at the same time, orthology can also help identify split genes (49). Furthermore, as discussed above, orthology combined with synteny information or integrated across multiple species in hierarchical groups can also uncover quality problems with the data.

One thing however seems certain: as the pace of genome sequencing continues to accelerate, elucidating evolutionary relationships across different genes will remain the key to exploiting the richness of this data. OMA is thus likely to stay relevant.

## SUPPLEMENTARY DATA

Supplementary Data are available at NAR Online.

## References

[1] Fitch W.M. (1970). Distinguishing homologous from analogous proteins. Syst. Zool..

[2] Sonnhammer E.L.L., Koonin E.V. (2002). Orthology, paralogy and proposed classification for paralog subtypes. Trends Genet..

[3] Gabaldón T., Koonin E.V. (2013). Functional and evolutionary implications of gene orthology. Nat. Rev. Genet..

[4] Sonnhammer E.L.L., Gabaldón T., Sousa da Silva A.W., Martin M., Robinson-Rechavi M., Boeckmann B., Thomas P.D., Dessimoz C., the Quest for Orthologs consortium (2014). Big data and other challenges in the quest for orthologs. Bioinformatics.

[5] Altenhoff A.M., Dessimoz C., Anisimova M (2012). Inferring orthology and paralogy. Evolutionary Genomics. Methods in Molecular Biology.

[6] Powell S., Forslund K., Szklarczyk D., Trachana K., Roth A., Huerta-Cepas J., Gabaldón T., Rattei T., Creevey C., Kuhn M. (2014). eggNOG v4.0: nested orthology inference across 3686 organisms. Nucleic Acids Res..

[7] Flicek P., Ahmed I., Amode M.R., Barrell D., Beal K., Brent S., Carvalho-Silva D., Clapham P., Coates G., Fairley S. (2013). Ensembl 2013. Nucleic Acids Res..

[8] Östlund G., Schmitt T., Forslund K., Köstler T., Messina D.N., Roopra S., Frings O., Sonnhammer E.L.L. (2010). InParanoid 7: new algorithms and tools for eukaryotic orthology analysis. Nucleic Acids Res..

[9] Uchiyama I., Mihara M., Nishide H., Chiba H. (2012). MBGD update 2013: the microbial genome database for exploring the diversity of microbial world. Nucleic Acids Res..

[10] Waterhouse R.M., Tegenfeldt F., Li J., Zdobnov E.M., Kriventseva E.V. (2013). OrthoDB: a hierarchical catalog of animal, fungal and bacterial orthologs. Nucleic Acids Res..

[11] Chen F., Mackey A.J., Stoeckert C.J., Roos D.S. (2006). OrthoMCL-DB: querying a comprehensive multi-species collection of ortholog groups. Nucleic Acids Res..

[12] Mi H., Muruganujan A., Thomas P.D. (2013). PANTHER in 2013: modeling the evolution of gene function, and other gene attributes, in the context of phylogenetic trees. Nucleic Acids Res..

[13] Huerta-Cepas J., Capella-Gutiérrez S., Pryszcz L.P., Marcet-Houben M., Gabaldón T. (2014). PhylomeDB v4: zooming into the plurality of evolutionary histories of a genome. Nucleic Acids Res..

[14] Proost S., Van Bel M., Sterck L., Billiau K., Van Parys T., Van de Peer Y., Vandepoele K. (2009). PLAZA: a comparative genomics resource to study gene and genome evolution in plants. Plant Cell.

[15] Altenhoff A.M., Schneider A., Gonnet G.H., Dessimoz C. (2011). OMA 2011: orthology inference among 1000 complete genomes. Nucleic Acids Res..

[16] Altenhoff A.M., Dessimoz C. (2009). Phylogenetic and functional assessment of orthologs inference projects and methods. PLoS Comput. Biol..

[17] Afrasiabi C., Samad B., Dineen D., Meacham C., Sjölander K. (2013). The PhyloFacts FAT-CAT web server: ortholog identification and function prediction using fast approximate tree classification. Nucleic Acids Res..

[18] Boeckmann B., Robinson-Rechavi M., Xenarios I., Dessimoz C. (2011). Conceptual framework and pilot study to benchmark phylogenomic databases based on reference gene trees. Brief. Bioinform..

[19] Linard B., Thompson J.D., Poch O., Lecompte O. (2011). OrthoInspector: comprehensive orthology analysis and visual exploration. BMC Bioinformatics.

[20] Roth A.C.J., Gonnet G.H., Dessimoz C. (2008). Algorithm of OMA for large-scale orthology inference. BMC Bioinformatics.

[21] Dessimoz C., Boeckmann B., Roth A.C.J., Gonnet G.H. (2006). Detecting non-orthology in the COGs database and other approaches grouping orthologs using genome-specific best hits. Nucleic Acids Res..

[22] Altenhoff A.M., Gil M., Gonnet G.H., Dessimoz C. (2013). Inferring hierarchical orthologous groups from orthologous gene pairs. PLoS One.

[23] Ashburner M., Ball C.A., Blake J.A., Botstein D., Butler H., Michael Cherry J., Davis A.P., Dolinski K., Dwight S.S., Eppig J.T. (2000). Gene ontology: tool for the unification of biology. The Gene Ontology Consortium. Nat. Genet..

[24] Dimmer E.C., Huntley R.P., Alam-Faruque Y., Sawford T., O'Donovan C., Martin M.J., Bely B., Browne P., Chan W.M., Eberhardt R. (2012). The UniProt-GO Annotation database in 2011. Nucleic Acids Res..

[25] Skunca N., Bošnjak M., Kriško A., Panov P., Džeroski S., Smuc T., Supek F. (2013). Phyletic profiling with cliques of orthologs is enhanced by signatures of paralogy relationships. PLoS Comput. Biol..

[26] Kersey P.J., Lawson D., Birney E., Derwent P.S., Haimel M., Herrero J., Keenan S., Kerhornou A., Koscielny G., Kähäri A. (2010). Ensembl Genomes: extending Ensembl across the taxonomic space. Nucleic Acids Res..

[27] Monaco M.K., Stein J., Naithani S., Wei S., Dharmawardhana P., Kumari S., Amarasinghe V., Youens-Clark K., Thomason J., Preece J. (2014). Gramene 2013: comparative plant genomics resources. Nucleic Acids Res..

[28] Rouard M., Guignon V., Aluome C., Laporte M.-A., Droc G., Walde C., Zmasek C.M., Périn C., Conte M.G. (2011). GreenPhylDB v2.0: comparative and functional genomics in plants. Nucleic Acids Res..

[29] Van Bel M., Proost S., Wischnitzki E., Movahedi S., Scheerlinck C., Van de Peer Y., Vandepoele K. (2012). Dissecting plant genomes with the PLAZA comparative genomics platform. Plant Physiol..

[30] Rensing S.A., Lang D., Zimmer A.D., Terry A., Salamov A., Shapiro H., Nishiyama T., Perroud P.-F., Lindquist E.A., Kamisugi Y. (2008). The Physcomitrella genome reveals evolutionary insights into the conquest of land by plants. Science.

[31] Marcussen T., Sandve S.R., Heier L., Spannagl M., Pfeifer M., Jakobsen K.S., Wulff B.B.H., Steuernagel B., Mayer K.F.X., International Wheat Genome Sequencing Consortium (2014). Ancient hybridizations among the ancestral genomes of bread wheat. Science.

[32] International Wheat Genome Sequencing Consortium (IWGSC) (2014). A chromosome-based draft sequence of the hexaploid bread wheat (Triticum aestivum) genome. Science.

[33] Luo M.C., Deal K.R., Akhunov E.D., Akhunova A.R., Anderson O.D., Anderson J.A., Blake N., Clegg M.T., Coleman-Derr D., Conley E.J. (2009). Genome comparisons reveal a dominant mechanism of chromosome number reduction in grasses and accelerated genome evolution in Triticeae. Proc. Natl Acad. Sci. U.S.A..

[34] Akhunov E.D., Sehgal S., Liang H., Wang S., Akhunova A.R., Kaur G., Li W., Forrest K.L., See D., Simková H. (2013). Comparative analysis of syntenic genes in grass genomes reveals accelerated rates of gene structure and coding sequence evolution in polyploid wheat. Plant Physiol..

[35] Choulet F., Alberti A., Theil S., Glover N., Barbe V., Daron J., Pingault L., Sourdille P., Couloux A., Paux E. (2014). Structural and functional partitioning of bread wheat chromosome 3B. Science.

[36] McHale L., Tan X., Koehl P., Michelmore R.W. (2006). Plant NBS-LRR proteins: adaptable guards. Genome Biol..

[37] Lagercrantz U., Axelsson T. (2000). Rapid evolution of the family of CONSTANS LIKE genes in plants. Mol. Biol. Evol..

[38] Geddy R., Brown G.G. (2007). Genes encoding pentatricopeptide repeat (PPR) proteins are not conserved in location in plant genomes and may be subject to diversifying selection. BMC Genomics.

[39] Byrne K.P., Wolfe K.H. (2005). The Yeast Gene Order Browser: combining curated homology and syntenic context reveals gene fate in polyploid species. Genome Res..

[40] Louis A., Muffato M., Roest Crollius H. (2013). Genomicus: five genome browsers for comparative genomics in eukaryota. Nucleic Acids Res..

[41] Steinbach D., Alaux M., Amselem J., Choisne N., Durand S., Flores R., Keliet A.-O., Kimmel E., Lapalu N., Luyten I. (2013). GnpIS: an information system to integrate genetic and genomic data from plants and fungi. Database.

[42] Carrigan M.A., Uryasev O., Davis R.P., Zhai L., Hurley T.D., Benner S.A. (2012). The natural history of class I primate alcohol dehydrogenases includes gene duplication, gene loss, and gene conversion. PLoS One.

[43] Schmitt T., Messina D.N., Schreiber F., Sonnhammer E.L.L. (2011). Letter to the editor: SeqXML and OrthoXML: standards for sequence and orthology information. Brief. Bioinform..

[44] De Bie T., Cristianini N., Demuth J.P., Hahn M.W. (2006). CAFE: a computational tool for the study of gene family evolution. Bioinformatics.

[45] Milinkovitch M.C., Helaers R., Depiereux E., Tzika A.C., Gabaldón T. (2010). 2X genomes–depth does matter. Genome Biol..

[46] Bailey J.A., Eichler E.E. (2006). Primate segmental duplications: crucibles of evolution, diversity and disease. Nat. Rev. Genet..

[47] Szalkowski A., Ledergerber C., Krähenbühl P., Dessimoz C. (2008). SWPS3 - fast multi-threaded vectorized Smith-Waterman for IBM Cell/B.E. and x86/SSE2. BMC Res. Notes.

[48] Wittwer L.D., Piližota I., Altenhoff A.M., Dessimoz C. (2014). Speeding up all-against-all protein comparisons while maintaining sensitivity by considering subsequence-level homology. PeerJ.

[49] Dessimoz C., Zoller S., Manousaki T., Qiu H., Meyer A., Kuraku S. (2011). Comparative genomics approach to detecting split-coding regions in a low-coverage genome: lessons from the chimaera Callorhinchus milii (Holocephali, Chondrichthyes). Brief. Bioinform..

